# Epac1 Is Crucial for Maintenance of Endothelial Barrier Function through A Mechanism Partly Independent of Rac1

**DOI:** 10.3390/cells9102170

**Published:** 2020-09-25

**Authors:** Alexander García-Ponce, Katharina Schuster, Stein-Ove Døskeland, Rolf K. Reed, Fitz-Roy E. Curry, Jens Waschke, Mariya Y. Radeva

**Affiliations:** 1 Chair of Vegetative Anatomy, Faculty of Medicine, Ludwig-Maximilians-University (LMU) Munich, Pettenkoferstraße 11, D-80336 Munich, Germany; Alexander.Garcia@med.uni-muenchen.de (A.G.-P.); katharinaschuster92@yahoo.de (K.S.); jens.waschke@med.uni-muenchen.de (J.W.); 2 Department of Biomedicine, University of Bergen, 5009 Bergen, Norway; stein.ove.doskeland@gmail.com (S.-O.D.); Rolf.Reed@uib.no (R.K.R.); 3 Centre for Cancer Biomarkers, University of Bergen, 5020 Bergen, Norway; 4 Department of Physiology and Membrane Biology, University of California Davis, Davis, CA 95616, USA; fecurry@ucdavis.edu

**Keywords:** cAMP, endothelial barrier, Epac1, Rho GTPases

## Abstract

Epac1 (exchange protein activated by cAMP) stabilizes the endothelial barrier, but detailed studies are limited by the side effects of pharmacological Epac1 modulators and transient transfections. Here, we compare the key properties of barriers between endothelial cells derived from wild-type (WT) and Epac1-knockout (KO) mice myocardium. We found that KO cell layers, unlike WT layers, had low and cAMP-insensitive trans-endothelial resistance (TER). They also had fragmented VE-cadherin staining despite having augmented cAMP levels and increased protein expression of Rap1, Rac1, RhoA, and VE-cadherin. The simultaneous direct activation of Rac1 and RhoA by CN04 compensated Epac1 loss, since TER was increased. In KO-cells, inhibition of Rac1 activity had no additional effect on TER, suggesting that other mechanisms compensate the inhibition of the Rac1 function to preserve barrier properties. In summary, Epac1 is crucial for baseline and cAMP-mediated barrier stabilization through mechanisms that are at least partially independent of Rac1.

## 1. Introduction

The aim of the present study was to further investigate the cAMP-dependent mechanisms stabilizing the intercellular junctions between vascular endothelium. Disruption of endothelial barrier integrity is a hallmark of many pathological disorders such as excessive edema and sepsis [1,2]. The specific focus was the role of the exchange protein activated by cAMP-(Epac1)-dependent pathways. These investigations were designed as follow-up research in Epac1^−/−^ (Epac1-knockout (KO)) mice that increased the basal permeability to high and low molecular weight tracers in different microvascular beds such as in the skin, small and large intestine, and adipose tissue. Their baseline microvascular permeability failed to drop further when exposed to atrial natriuretic peptide or histamine, and the permeability reduction in response to the phosphodiesterase 4 (PDE4) inhibitor Rolipram was blunted [3,4]. Moreover, these animals exhibited a longer bleeding time in vivo [5]. Interestingly, no increased heart permeability [3] or changes in heart size were observed, however, cardiac function from the Epac1-KO mice was compromised [6]. A key feature of the experimental design is that the endothelial cells used in the present study were isolated and cultured from the same Epac1-deficient mice and their respective wild-type (WT) control littermates, as that used for the whole organ permeability measurements. This approach overcomes some of the limitations of in vitro studies that rely on pharmacological manipulation and transfection to diminish the effects of Epac1. The present cells not only allow the effect of Epac1-deficiency to be investigated more directly, but also enable an appraisal of the role of PKA alone in mediating cAMP effects on barrier integrity.

The two main types of cell–cell contacts between endothelial cells are adherens junctions (AJs) and tight junctions (TJs). TJs formed by claudins and occludin seal the intercellular cleft between neighboring cells to limit paracellular permeability. The VE-cadherin-based AJs provide mechanical adhesive strength between opposing cells. Both TJs and AJs are tethered to the actin cytoskeleton through adaptor proteins like zonula occludens (ZOs) and members of the catenin family [1,7]. The TJ and AJ tethering is regulated by small Rho GTPases like Rac1. The latter can be stimulated either by engaging VE-cadherin to a forming junction [8,9,10], or by outside-in signaling, triggered by extracellular spinghosine-1-phosphate (S1P) [11] or angiopoietin-1 (Ang-1) [12,13], as well as by an intracellular cAMP increase. Cyclic AMP can trigger Rac1, either through protein kinase A (PKA) or Epac1, via guanosine nucleotide exchange factors (GEFs) like T-cell lymphoma invasion metastasis 1 (Tiam1) and Vav2, and thus, modulate its GTPase activity, which consequently leads to the regulation of various downstream signaling pathways.

Epac1 was discovered as a cAMP-binding protein responsible for PKA-independent Rap1 activation [14,15], which is linked to enhancement of the endothelial barrier function during inflammation [16,17]. Rap1 activity is shown to regulate junctional adhesion through actions on TJ, while Rap1 inhibition can affect the localization of the TJ marker ZO-1 in endothelial cells [18]. Furthermore, Epac1 activation via 007 leads to junctional reorganisation between neighboring cells, as demonstrated by the strengthened VE-cadherin-dependent junctional integrity in vitro and in vivo [19,20], and increased claudin-5-mediated TJ formation in vitro [21]. Other studies also reported that Rap1 might balance the dynamic of AJs by differentially regulating both, i.e., the Cdc42/MRK/NM-II- and the Rho/ROCK signaling pathways. Thus, it was shown that Epac1-mediated Rap1 activation via FGD5 can induce Cdc42-MRCK-NMII signaling, and thereby, enhance the endothelial barrier function. Furthermore, Rap1 probably via its effector Krit1, could also suppress the Rho/ROCK/NM-II pathway, activation of which indeed might lead to endothelial barrier dysfunction [22]. In this line of thinking, it was reported that Krit1, which binds to and is regulated by Rap1, might establish interaction with CCM2. Both, Krit1 and CCM2 were shown to be negative regulators of RhoA and its effector ROCK, thus, limiting endothelial barrier permeability in vitro and vascular leak [23]. Similarly, other studies where vascular smooth muscle [24] and human umbilical vein endothelial cells (HUVEC) were tested also manifested that activation of Epac1 led to the attenuation of RhoA activity [25]. Correspondingly, Post and colleagues revealed that activation of Rap1 induces translocation of its effectors Rasip1 and Radil to the plasma membrane, as the latter form a complex with Rho GTPase activating protein ArhGAP29. The relocation of these proteins provoked the formation of a multimeric protein complex required for Rap1-induced inhibition of Rho signaling, and increased the endothelial barrier function [26]. The correlation between Rap1-RhoA is further supported by a study from Birukova et al. [27], where siRNA-mediated depletion of Rap1 in human pulmonary endothelial cells led to delayed endothelial barrier recovery associated with prolonged Rho-kinase-dependent phosphorylation of myosin light chain phosphatase (MYPT) and myosin light chain (MLC). In contrast with the above listed reports, there are studies available where the opposite effect of Rap1 activation towards RhoA was evident. Thus, it was demonstrated that long-term stimulation of Epac1 can promote Rap1-dependent RhoA activity in smooth muscle cells, leading to filamentous (F)-actin stabilization via increased activity of ROCK [28]. Therefore, it is suggested that Epac1-dependent barrier stabilization is achieved not only via modulation of junctional integrity but also by rearrangement of cortical actin to the cell periphery, as well as by cross-talk between GTPases. The aforementioned studies indicate that Epac1 is a central player working through multiple mechanisms to promote cAMP-mediated endothelial barrier stabilization. Our current study describes the establishment of Epac1-deficient endothelial cells and their use to elucidate in more detail the role of Epac1 in cAMP-mediated regulation of Rho GTPases and endothelial barrier homeostasis.

## 2. Materials and Methods

### 2.1. Cell Culture and Isolation of Endothelial Cells

Mouse microvascular myocardial endothelial cell (MyEnd) lines were generated and immortalized, as described elsewhere [29,30]. In brief, myocardial tissues were derived from either wildtype (Epac1-WT) or Epac1 knockout (Epac1-KO)/RapGef3^−/−^ newborn pups. The latter showed deletion of exon 7 coding for the only functional cAMP domain of Epac1. The Epac1 null mice also showed a frame shift mutation distal to the deletion, which aimed to prevent the expression of any C-terminal Epac1 fragments through unscheduled protein synthesis (see Ref [3], for details). The extracted tissues were chopped into small pieces. The resulting tissue fragments were consequently digested with Trypsin-Collagenase A solution (0.05% Trypsin Biochrom, Berlin, Germany, and 0.02 % Collagenase A, Roche Diagnostics, Mannheim, Germany). Digestion was stopped by adding equal volume of ice-cold Buffer A (153 mM NaCl, 5.6 mM KCl, 2.3 mM CaCl_2_x2H_2_O, 2.6 mM MgCl_2_x6H_2_O, 15 mM HEPES, 1% BSA, Sigma Aldrich Chemie GmbH, Taufkirchen, Germany). The arising cell suspensions were briefly centrifuged. Subsequently, the cell pellets were resuspended in culture medium and seeded onto gelatin-coated culture dishes. To immortalize the cells, 24 h after isolation, the adherent cells were treated with Polyoma virus middle T antigen, secreted by GP+E-86 Neo (GPENeo) fibroblast. Thus, endothelial cell growth was preferentially enhanced compared to the non-endothelial cells. As a result, a homogenous monolayer of endothelial cells was obtained after 4 to 6 weeks of culturing. Similar to previous studies [29,31,32], the endothelial phenotype was confirmed by testing the cells for expression of VE-cadherin and von Willebrand factor through immunostaining and Western Blot analyses.

The immortalized MyEnd cell lines were cultured at 37 °C, in a humidified atmosphere of 5% CO_2_ and grown in Dulbecco’s Modified Eagle’s medium (DMEM, Gibco-ThermoFisher, Munich, Germany, Cat# 41966-029), supplemented with 50 U/mL Penicillin G/Streptomycin (Sigma Aldrich Chemie GmbH, Taufkirchen, Germany), and 10% Fetal Calf Serum (FCS, Biochrom, Berlin, Germany, Cat# S0115/0247X). The presence or lack of Epac1 was determined by PCR analysis of genomic DNA and Western blot analysis of protein extracts.

### 2.2. Ethical Approval

All experiments were approved by the Ethics committee of the Regierung von Oberbayern, Germany (Az. 55.2-2532.Vet_02-14-139) and were performed in accordance with the relevant guidelines and regulations.

### 2.3. Antibodies and Test Reagents

Increase of intracellular cAMP levels was achieved by application of 5 µM Forskolin (F) (Sigma Aldrich Chemie GmbH, R6520, Taufkirchen, Germany), an adenylyl cyclase (AC) activator, and 10 µM Rolipram (R), (Sigma Aldrich Chemie GmbH, F6886, Taufkirchen, Germany), a phosphodiesterase 4 (PDE4) inhibitor (Sigma Aldrich Chemie GmbH, R6520, Taufkirchen, Germany) for 1 h. Predominant activation of Epac1/Rap1-signaling was accomplished by incubation with 200 µM of 007 (8-pCPT-2′-O-Me-cAMP, Biolog, Cat# C041-05, Bremen, Germany), for 1 h. Preferential modulation of PKA signaling was done by administration of 100 µM 6Bnz-cAMP (Biolog, Cat# B0009, Bremen, Germany), for 6 h. For direct activation of small GTPases, RhoA/Rac1/Cdc42 cells were incubated with Rho/Rac/Cdc42 Activator I (Cytoskeleton Inc, Cat#CN04, Denver, CO, U.S.A.), at a final concentration of 0.25 μg/mL for 2 h. To inhibit Rac1 activity, the cell monolayers were treated for at least 2 h with 50 µM of the specific pharmacological blocker EHT-1864 (Tocris, Cat#3872, Bristol, U.K.).

The following antibodies were used—goat anti-VE-cadherin (Santa Cruz Biotechnology Inc., Sc. 6458, Dallas, TX, USA); mouse anti-α-tubulin (Abcam, ab7291, Cambridge, UK), rabbit anti-PDE4-D (Antikorper-online.de, ABIN2404474, Aachen, Germany), mouse anti-RhoA, clone 7F1.E5 (Cytoskeleton Inc., Cat# ARH04-5, Denver, CO, USA), mouse anti-Rac1 (BD Biosciences, Cat# 610651, Franklin Lakes, NJ, USA), rabbit anti-Rap1 (Merck-Sigma Aldrich Chemie GmbH, 17-321, Taufkirchen, Germany), rabbit anti-Epac1 (Abcam, ab109415, Cambridge, UK), mouse anti-Epac2 5B1 (Cell Signaling, #4156, Leiden, The Netherlands), and rabbit anti-pVASP/VASP IG731 (ImmunoGlobe® Antikörpertechnik GmbH, 0012-02, Himmelstadt, Germany).

### 2.4. Western Blots

Confluent MyEnd monolayers were washed with ice cold PBS and then lysed with SDS-lysis buffer (25 mM HEPES, 2 mM EDTA, 25 mM NaF and 1% SDS, pH 7.6), to which a cOmplete™ protease inhibitor cocktail (Roche Diagnostics, #11697498001, Mannheim, Germany) was routinely added. After sonication, protein concentration of the lysates was determined by the Pierce BCA protein assay kit (Thermo Fischer Scientific, Waltham, Germany). Equal amount of protein was loaded on polyacrylamide gels and subsequently separated by electrophoresis. Western blotting was performed according to the standard procedures. Membranes were blocked with 5% non-fat milk in TBS-0.1% Tween and probed overnight at 4 °C with primary antibodies, specific for the proteins of interest. After incubation with the respective species-specific Horseradish Peroxidase (HRP)-conjugated secondary antibody, the membranes were embedded in enhanced chemiluminescence (ECL) Western Blotting detection reagents and consequently developed using the Amersham Imager 600 (AI600, GE Healthcare, Munich, Germany). Densitometric analysis of the signal intensity from each specific protein band was performed by ImageJ, as described elsewhere [33].

### 2.5. Transendothelial Electrical Resistance Measurements

To monitor the dynamics of cell–cell contacts in real time and thereby the endothelial barrier integrity of confluent cell monolayers, the ECIS Z Theta system (Applied Biophysics Inc, Troy, NYC, U.S.A.) was used, as described previously [34]. In brief, MyEnd cells were seeded in 0.5% gelatine pre-coated 8W10E gold microarray electrodes (Ibidi, Martinsried, Germany). Prior to the start of the experiment, the medium was exchanged and the electrodes were mounted into the ECIS system’s array holders, where they were pre-stabilized and equilibrated at 37 °C in a humidified atmosphere of 5% CO_2_, for at least 1 h. Experiments were performed in the Multifrequency (MTF) mode, however, all ECIS data presented here was at 4000 Hz—the optimal frequency for endothelial cells determined by us and others [34].

### 2.6. Immunofluorescence and Densitometric Measurements

Cells were seeded on 0.5% gelatine pre-coated 12 mm glass cover slips and grown to confluence. The monolayers were exposed to various test reagents and fixed with 4% paraformaldehyde for 20 min at room temperature. Cells were then permeabilized with 0.5% Triton-X-100 in PBS for 5 min and immunolabeling of the proteins of interest was accomplished using the standard procedures [35,36].

VE-cadherin protein distribution was quantified using the Plot Profile tool from the ImageJ software, as previously described [36]. In brief, to determine the intensity of the signal, rectangular marquees were drawn orthogonal to the membrane; regions within and around the membrane were incorporated. More than 25 randomly selected areas of plasma membranes from images collected from four independent experiments, were evaluated. The intensity distribution of the protein of interest was recorded for each rectangle. The peak of the bell-shaped curve represents the highest value defining the middle of the cell membrane. The equal number of points before and after the peak denotes the respective shoulders of the generated curve. Approximately, the first and the last 0.5 µm from each shoulder correspond to areas around the membrane, i.e., the intracellular zone, while the rest of the shoulder represents the space within the membrane. The data are presented either as raw values or normalized to the intensity of the starting point within the intracellular region from WT-controls, which was set to one (1).

### 2.7. Rac1 and RhoA GLISA Measurements

Commercially available G-protein ELISA assays (Cytoskeleton, Denver, CO, U.S.A.) were utilized to measure the intracellular concentration of active, GTP-bound Rac1 and RhoA proteins, in both control cells and cells treated with the indicated pharmacological modulators. After incubation, the cell monolayers were lysed and processed, according to the manufacturer’s instructions. Absorbance measurements were performed at 490 nm, using a TECAN, Infinite 200 PRO microplate reader (Tecan Deutschland GmbH, Crailsheim, Germany).

### 2.8. Non-Radioactive Rap1 Pull-Down Activity Assay

MyEnd cells were seeded on gelatin-coated T75 cell culture flasks. Confluent cell sheets were treated with either DMSO (Vehicle) or F/R for 1 h. Afterwards, the cells were washed with ice-cold PBS and immediately lysed with Rap1 Activation Lysis Buffer provided with the Rap1 Activation Assay Kit (Merck Chemicals GmbH, Cat# 17-321, Darmstadt, Germany). Lysates were cleared from the cell debris by centrifugation at 14,000 rfc, 4 °C for 5 min. Samples were snap frozen and stored at −80 °C, a few days before processing. Protein concentration was determined with Pierce BCA protein assay kit, as previously described. A total protein amount of 1 mg was used for each pull-down. The assay was performed according to the manufacturer’s instructions.

### 2.9. Quantitative Determination of Cyclic AMP

Cellular cAMP levels, in the control or treated samples, were measured by the cAMP enzyme linked immunosorbent assay (Sigma Aldrich Chemie GmbH, Taufkirchen, Germany), following the manufacturer’s instructions.

### 2.10. PDE Activity Assay

Total PDE activity was quantified with the commercially available kit (BioVision, Cat. # K927, Milpitas, CA, U.S.A.). In brief, confluent endothelial cell monolayers grown on gelatine pre-coated 10 cm petri dishes were lysed with 100 μL ice-cold PDE Assay Lysis Buffer. Shortly afterwards, the lysates were incubated on ice for 10 min and subsequently centrifuged at 10,000 rfc for an additional 10 min at 4 °C. Protein concentration in each sample was determined, as previously mentioned. Equal amount of proteins was used for the assay, and the total PDE activity was determined, following the manufacturer’s instructions.

### 2.11. PCR Analysis

cDNA generated from the total RNA extracted from the WT and Epac1-KO cells was used as a template for amplification of the Epac1 (378 base pairs (bp)) gene fragment. Additionally, junctional VE-cadherin (139 bp) was analyzed. Equal loading was confirmed by the expression of beta-2-microtubulin (B2M) gene, (292 bp). In the presence of genomic DNA contamination, the B2M primer pair would amplify an additional PCR product of size 788 bp. The latter amplicon was not detected in any of the samples tested. All primers used in the assay are listed in Table 1.

### 2.12. Data Analysis and Statistic

Unpaired *t*-test was used to compare the differences between the two groups. When three or more groups were analyzed, two-way ANOVA, followed by either Tukey’s or Sidak multiple comparison test was applied. Data are presented as a mean ± SEM. All *p*-values equal or less than 0.05 were considered to be statistically significant. 

## 3. Results

### 3.1. Epac1 Contributes to Baseline Endothelial Barrier Function and Is Crucial for the cAMP-Mediated Barrier Stabilization

**Figure 1:**
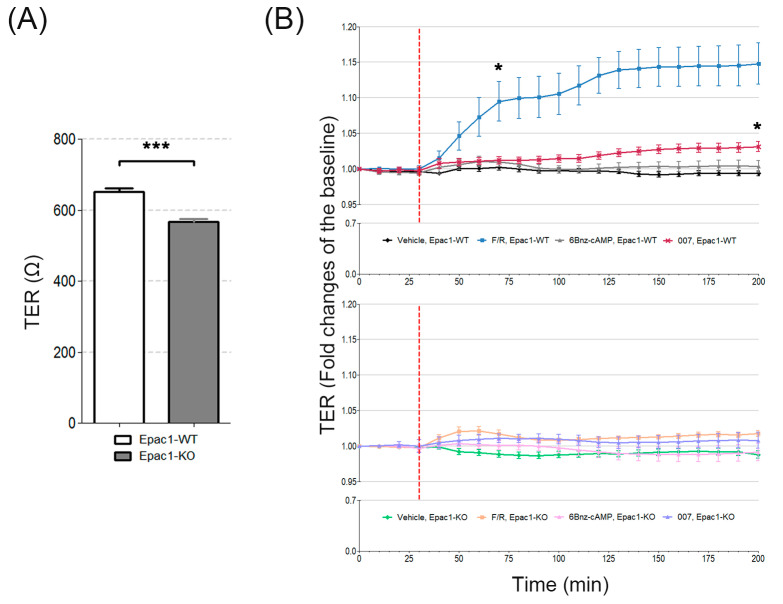
TER from MyEnd monolayers under basal and after the cAMP stimulated conditions. (**A**) Bar diagram representing the average basal transendothelial resistance monitored by ECIS in both WT and Epac1-KO cell lines. Data were collected 200 min after the measurement was initiated. (**B**) Barrier function, over time, from the control and treated (as indicated) WT and Epac1-KO cell monolayers assessed by TER. The segmented red line denotes the time of mediator application. Left asterisk indicates statistically significant difference between F/R stimulated WT cells and the corresponding Vehicle controls. Right asterisk denotes differences between 007 treated WT cells and the respective control monolayers. N = 4–5; n ≥ 8; * *p* ≤ 0.05, *** *p* ≤ 0.001.

To judge the importance of Epac1 for cell monolayer integrity in vitro, the transendothelial electrical resistance (TER) was determined in the MyEnd cell lines derived from WT and Epac1-null C57BL/6J mice. Under the basal/untreated conditions, the average resistance was lower (573 ± 9.3 Ω) in confluent Epac1-KO MyEnd cell monolayers than in the WT cell monolayers (721 ± 58.7 Ω) (Figure 1A). Modeling of TER data was performed using the ECIS software. These calculations confirmed that Epac1-KO monolayers had indeed reduced barrier function (Supplementary Table S1).

In WT cells whose intracellular cAMP was increased by incubation with the AC activator, forskolin (F), and the PDE4 inhibitor, rolipram (R), the TER increased as expected [36,37]. TER increased rapidly by more than 10–15% in the Epac1-WT cells. Similar to the observations in intact mice [3], the Epac1-KO cells responded to F/R much less than the WT cells. Treatment of cells with the Epac1 activator 007 led to a minor, but significant, elevation in TER, only in Epac1-WT cells (Figure 1B). Our observations were in line with the previous report from Kooistra and colleagues [19], which showed that 007-mediated Epac1 activation reduced the FITC-labeled dextran permeability of WT cells between 30–50%, while this effect was completely abolished upon siRNA-mediated Epac1 depletion. The specific PKA activator, 6Bnz-cAMP, failed to affect TER in either cell line (Figure 1B). Graph representing the original (raw) data is provided in Supplementary Figure S1 (data from modeling is available in Supplementary Table S1). PKA activity was verified by detection of VASP at Ser157 with the Western blot (Supplementary Figure S2A). We concluded that in our cellular model, Epac1 was the dominant mediator of cAMP-induced endothelial cell barrier tightening.

### 3.2. Epac1 Mediates the Recruitment of VE-Cadherin to AJs upon cAMP Elevation

**Figure 2:**
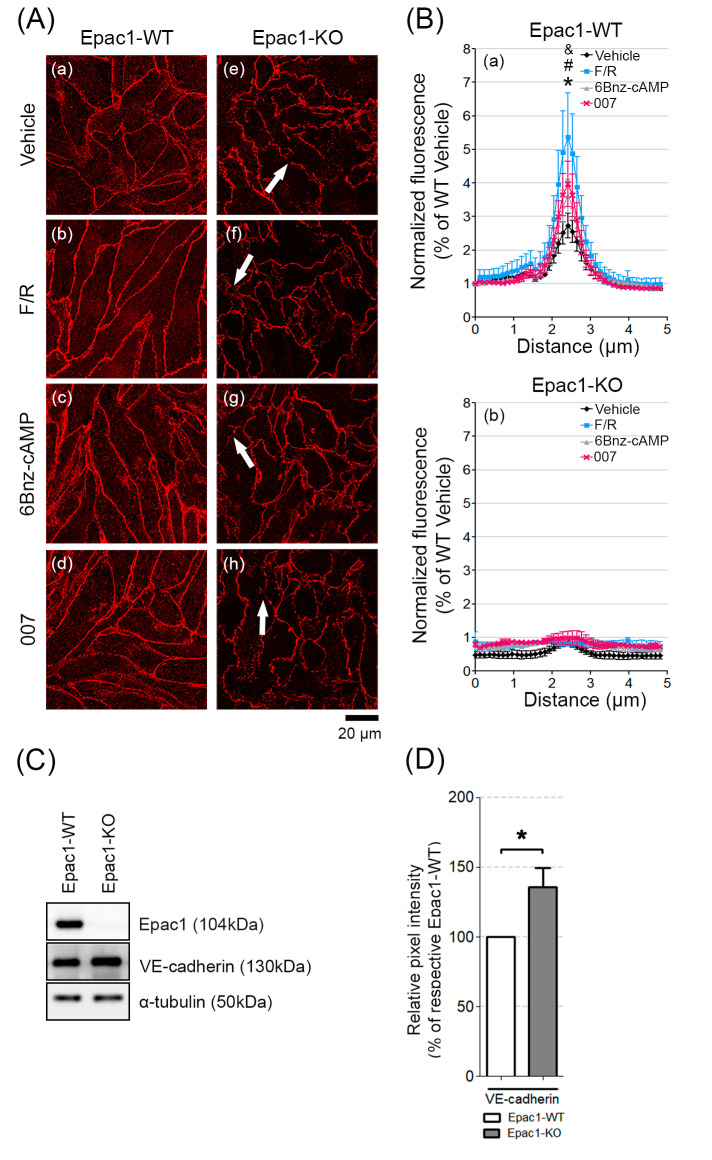
Distribution and basal protein levels of VE-cadherin in confluent MyEnd cell sheets. (**A**) Confluent MyEnd cell monolayers originating from WT (a–d) and Epac1-KO (e–h) mice were treated either with vehicle (DMSO) or with F/R (1 h), 007 (2 h), or 6-Bnz-cAMP (6 h). Arrows indicate VE-cadherin fragmentation among the neighbouring cells. (**B**) Bell-shaped curve representing the distribution of VE-cadherin signal intensity across cell borders. Normalized data collected from WT and Epac1-KO cell lines are presented. N = 4, n = 25; “*” denotes statistical significance for vehicle vs. F/R in WT cell monolayers; “#” for F/R vs. 6Bnz-cAMP; “&” for F/R vs. 007; *p* ≤ 0.05. (**C**) Western blot analysis of whole cell lysates for Epac1 and VE-cadherin. Equal loading validation was monitored with α-tubulin; N ≥ 10. (**D**) Respective densitometric measurements of the band intensity; N ≥ 10. Full-length blots can be found in the Supplementary Figure S3. Data are presented as mean ± SEM.

We next analyzed if the lack of Epac1 led to changes in the junctional structures. The WT cell monolayers had mostly contiguous VE-cadherin junctional staining, which became linear and augmented in response to treatment with either F/R, 6-Bnz-cAMP, or 007 (Figure 2A, a–d). Monolayers of cells lacking Epac1 had fragmented VE-cadherin membrane distribution under all tested conditions (Figure 2A, e–h, arrows). Quantification of the VE-cadherin signal intensity across the cell borders showed that only WT cells reacted with stronger membrane signal, in response to the various cAMP-inducing stimuli (Figure 2B, (a) for WT; (b) for Epac1-KO). However, only the increase mediated by F/R turned to be statistically significant when compared to the vehicle-treated cells. Thus, our data suggest that in our cell system, Epac1 appeared to be required for the cAMP-induced recruitment of VE-cadherin to cell junctions.

### 3.3. Loss of Epac1 Affects the Expression of the Junctional Adhesion Protein VE-Cadherin 

It is known that Epac1 can regulate endothelial junctional integrity through VE-cadherin [19]. In addition, a member of the E-twenty-six (ETS) family of transcription factors, Elk3, was shown to regulate the VE-cadherin gene expression, since Elk3 downregulation resulted in increased VE-cadherin expression [38]. Moreover, it was reported that Epac1 can control transcription factor activity of another ETS family member—Elk1 [39], potentially modulating the protein expression. We therefore tested if deletion of Epac1 could alter VE-cadherin expression using RT-PCR and WB analyses.

We found no statistically significant difference of VE-cadherin mRNA expression between WT and Epac1-KO cells (Supplementary Figure S4A,B). Despite this, high VE-cadherin protein expression in Epac1-KO was observed, compared to WT cells (Figure 2C,D). Thus, the increased VE-cadherin protein levels in Epac1-KO cells might act as a compensatory response caused by Epac1 loss, however, it does not appear to be sufficient enough to stabilize the barrier.

### 3.4. The Expression and Activity of Rap1, Rac1, and RhoA in the WT and Epac1 Null Cells

**Figure 3:**
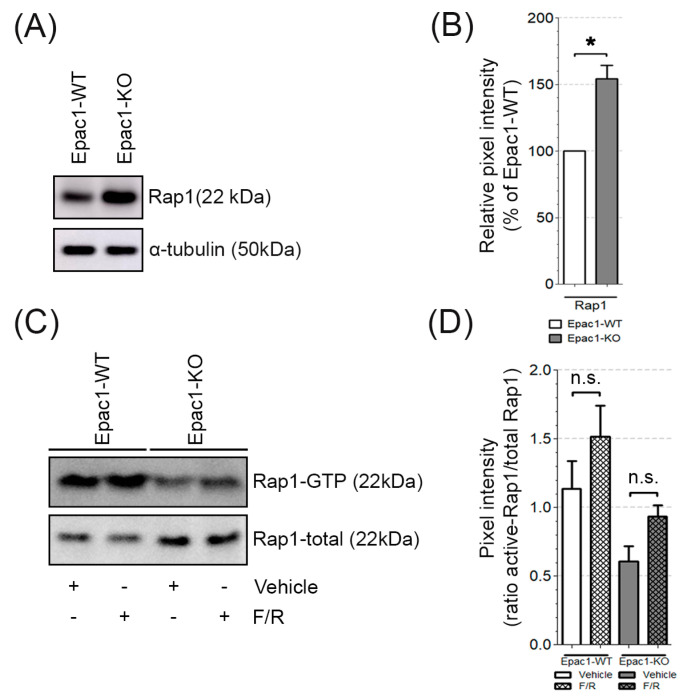
Basal and GTP-bound levels of Rap1. (**A**) Representative Western blot of total Rap1; α-tubulin was used as a loading control. N = 5, n ≥ 2. (**B**) Relative expression of Rap1 in Epac1-KO was compared to the respective expression in WT cells; * *p* ≤ 0.05. (**C**) Rap1 activation in WT and Epac1-KO cells, treated with either vehicle (DMSO) or F/R. The activity of Rap1 was assessed by pulldown assays; N = 4. (**D**) Densitometric measurements from the Rap1-GTP pull-downs were normalized to the respective total Rap1. “n.s.” denotes non-significant differences.

Under the resting state, cAMP signaling via Epac1 and its effector Rap1 appears to be dominant for tonic barrier maintenance in vivo [40]. Therefore, we analyzed how the loss of Epac1 affected the expression and activity of Rap1. The analysis disclosed that the Epac1-KO cells exhibited increased protein expression of the Epac1 effector Rap1 (Figure 3A,B), but showed a big trend towards decreased overall Rap1 activity in either of the conditions analyzed, as evidenced by the Rap1-GTP Pull-down assays (Figure 3C,D, original blots can be found in Supplementary Figure S5). These results indicate that other mechanisms could partially compensate the loss of Epac1. For this reason, we analyzed whether MyEnd cells also express Epac2. The latter was detected in both WT and Epac1-KO cells, an observation that was in contrast to other studies, where Epac2 was not expressed by other endothelial cell types [19]. Interestingly, the levels were higher in cells lacking Epac1 (Supplementary Figure S6A,B). 

**Figure 4:**
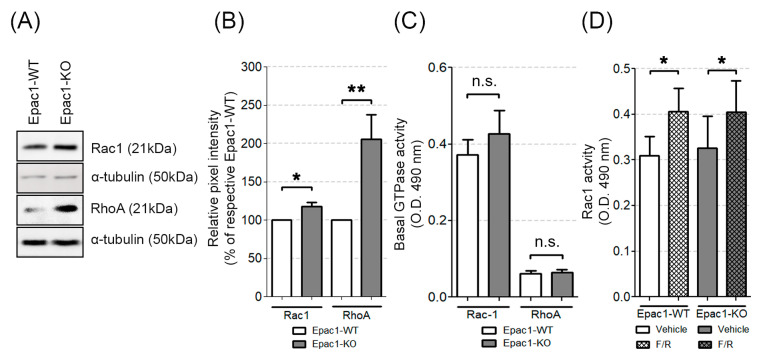
Basal protein levels and activation state of the small GTPases Rac1 and RhoA. (**A**) Western blot analysis of whole cell lysates for Rac1 and RhoA. Equal loading validation was monitored with α-tubulin; N ≥ 10. (**B**) Respective densitometric measurements of the band intensity. (**C**) Activity levels of basal Rac1 and RhoA assessed by G-LISA in WT and Epac1-KO cell lines; N ≥ 9. (**D**) Rac1 G-LISA activity measured in either DMSO or F/R treated WT and Epac1-KO cell lines; N = 8; * *p* ≤ 0.05, ** *p* ≤ 0.01; “n.s.” denotes non-significant differences between the analyzed groups.

The junctional organization and barrier integrity can, in addition to Rap1, be regulated by small Rho GTPases, which act by altering the actin cytoskeleton dynamics (see Introduction section for details). Therefore, we wanted to evaluate the impact of Epac1 on the protein expression and activation state of these GTPases in endothelial cells. We found that the Epac1-KO cells had increased basal Rac1 and RhoA protein expression (Figure 4A,B), but showed similar Rac1 and RhoA activity to the one detected in WT cells (Figure 4C, original WB images can be found in Supplementary Figure S3).

We conclude that the Epac1-deficient endothelial cells showed increased expression of Rap1, Rac1, and RhoA, but only the activity of Rap1 was decreased in both the control and the cAMP-stimulated conditions. Thus, in MyEnds, Epac1 is required for efficient Rap1 function but does not affect the activity of small Rho GTPases.

### 3.5. Epac1 Appears Not to Be Required for cAMP-Mediated Rac1 Activation

It is well known that Epac1 and its effector Rap1 are crucial for cAMP-induced tightening of endothelial junctions. We showed here that, in both cell lines, cAMP elevation led to an apparent increase in Rap1 activity, which was less prominent for the Epac1 deficient cells (Figure 3C,D). These results indicated that besides Epac1, other signaling molecules could trigger Rap1 activity. Rac1 activation might in part be mediated by Epac1 [34], and it could thus contribute to the barrier stabilizing effects of cAMP. We therefore compared the effect of F/R-induced cAMP increase on Rac1 activity in WT and Epac1-KO cells. We found increased Rac1 activation following F/R treatment both in WT and Epac1-KO cells (Figure 4D), suggesting that Epac1 was dispensable for the cAMP-mediated Rac1 activation in our endothelial cells.

**Figure 5:**
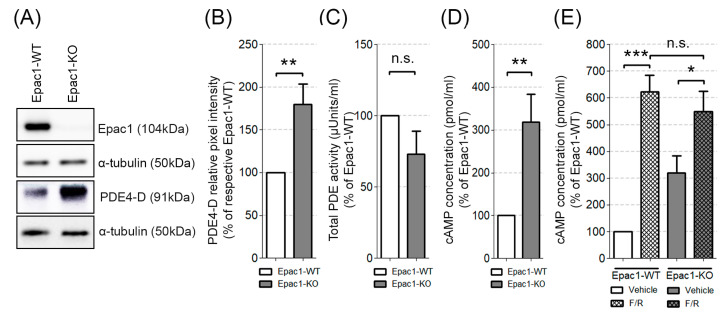
Expression of PDE4-D and intracellular levels of cAMP. (**A**) Western blot analysis of PDE4-D; α-tubulin was used as the loading control, N ≥ 5. (**B**) Densitometric evaluation of the PDE4-D band intensity, N ≥ 5. (**C**) Total basal PDE activity of Epac1-KO and the corresponding WT control cells, N = 4, n ≥ 8. (**D**) The basal intracellular cAMP levels in the WT and Epac1-KO endothelial cells; N ≥ 10. (**E**) The cAMP levels of the WT and KO cells subjected to F/R, N ≥ 5; * *p* ≤ 0.05, ** *p* ≤ 0.01, *** *p* ≤ 0.001; “n.s.” denotes non-significant differences between the analyzed groups.

Rampersad and colleagues found that PDE4-D can tether Epac1 in a macromolecular complex connected to the VE-cadherin mediated junctions. Moreover, they showed that either siRNA-mediated Epac1 knockdown or lack of PDE4-D, increased the endothelial barrier permeability [41]. We therefore tested whether Epac1 deficiency altered the expression or enzymatic activity of PDE4-D. We found that our Epac1 null endothelial cells showed increased basal expression of PDE4-D protein (Figure 5A,B, original blots can be found in Supplementary Figure S7), but no increase of total cellular PDE activity (Figure 5C). The basal total cellular cAMP levels were higher in Epac1-KO than the WT cells (Figure 5D), suggesting that the delocalization of PDE4-D might lower its efficiency. However, the exposure to F/R raised the cAMP concentration in either cell line. Thus, no striking difference of the cAMP content was detected between the WT and Epac1-KO cells (Figure 5E). In conclusion, the cells devoid of Epac1 had intact cellular cAMP levels and robust Rac1 activation, in response to F/R.

### 3.6. Simultaneous Direct Activation of Rac1 and RhoA Enhanced Barrier Function in the Absence of Epac1 

**Figure 6:**
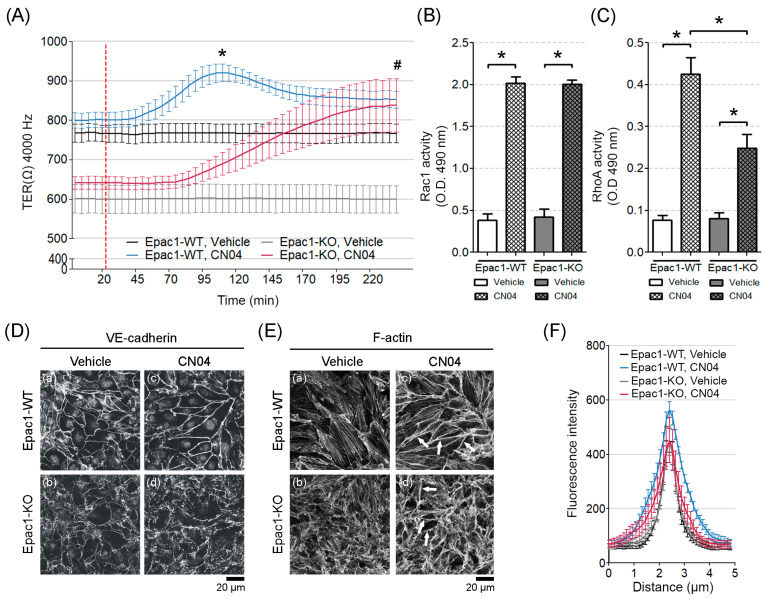
Effects of simultaneous activation of Rac1 and RhoA on TER, GTPase activation, VE-cadherin and F-actin distribution. (**A**) After achieving a stable TER, the cells monolayers were administered with a CN04 mediator (segmented red line). Measurements for the respective controls were performed in parallel. Barrier resistance was monitored; N = 4–5, n ≥ 8; “*” denotes statistical significance for the WT-Vehicle vs. WT-CN04; “#” for the KO-Vehicle vs. KO-CN04. (**B**) Rac1 G-LISA activation assay in WT and the Epac1-KO cell lines subjected to DMSO or CN04; N = 7; * *p* ≤ 0.05. (**C**) RhoA G-LISA activation assay in the WT and KO cell lines exposed to DMSO or CN04; N = 6; * *p* ≤ 0.05. (**D**) VE-cadherin immunostaining from the confluent control (a and b) or the CN04-treated (c and d) cell monolayers; N = 4. (**E**) Effect of Rho GTPases on F-actin remodeling in the control (a and b) and the CN04-treated (c and d) confluent cell sheets. Arrows indicate cortical actin; N ≥ 4. (**F**) The intensity of VE-cadherin staining was quantified by densitometric measurements; N = 4; n ≥ 25 cells per N.

Since the Epac1-KO cells could not respond to the cAMP-mediated barrier stabilization, despite proper Rac1 activation upon cAMP increase, we hypothesized that apart from Rac1, RhoA might be required to enhance the endothelial barrier function when Epac1 was absent. To test this, we used the synthetic Rho GTPases activator, CN04, to achieve the constitutive GTPases activity. Endothelial barrier behavior was monitored in real-time by ECIS. Both, WT and Epac1-KO confluent cell monolayers responded with an increased barrier resistance to treatment with 0.25 μg/mL CN04 for 2 h. In fact, the Epac1-KO cells achieved similar stable resistance as the control cells, after 2.5 h (Figure 6A), enduring for hours thereafter (data not shown). It therefore appeared that a synchronized direct activation of the Rho small GTPases could improve the endothelial barrier properties, independent of Epac1. Corresponding G-LISA experiments corroborated that both Rac1 and RhoA were activated by CN04 in the cell lines tested (Figure 6B,C), but the effect was significantly less for RhoA, in the cells lacking Epac1, suggesting that Epac1 might be required for proper RhoA activation. This was in accordance with our previous study in human dermal microvascular endothelial cells (HDMEC), where Rac1 and RhoA activities increased simultaneously, upon treatment with a CN04 analog, i.e., cytotoxic necrotizing factor-1 (CNF-1). Additionally, exposure of HDMEC to CNF-1 resulted in significantly reduced FITC-dextran flux and increased barrier integrity [42].

To know if the barrier effects of CN04 correlated with modulation of junctional protein localization, the confluent monolayers were immunostained for VE-cadherin. In either cell line, CN04 stimulation led to AJs remodeling, resulting in augmented VE-cadherin staining along the junctions. (Figure 6D, c–d). Quantification of the VE-cadherin signal intensity showed that CN04 not only increased the VE-cadherin signal, particularly in the Epac1-WT cells, but also led to thicker AJs, corresponding to a wider bell-shaped form (Figure 6F, blue curve). The latter effect was less prominent in the Epac1-KO cells (Figure 6F, red curve).

Sphingosine-1-phosphate (S1P) can strengthen the endothelial barrier by activation of RhoA and the ensuing F-actin accumulation juxtaposed with intercellular junctions [43]. We therefore studied if the RhoA pathway of barrier stabilization could be operative and depend on Epac1 in our endothelial cell lines. Thus, the distribution of F-actin by applying ALEXA-488-conjugated phalloidin to the confluent cell monolayers, was analyzed. We noted that the F-actin fibers appeared less organized in the Epac1-KO cells than in the WT cells, under basal conditions (Figure 6E, a and b). Incubation with CN04 (2 h) further increased the F-actin content at the junction-associated actin belt, more strongly in WT than in the Epac1 null cells (Figure 6E, c and d, arrows). 

**Figure 7:**
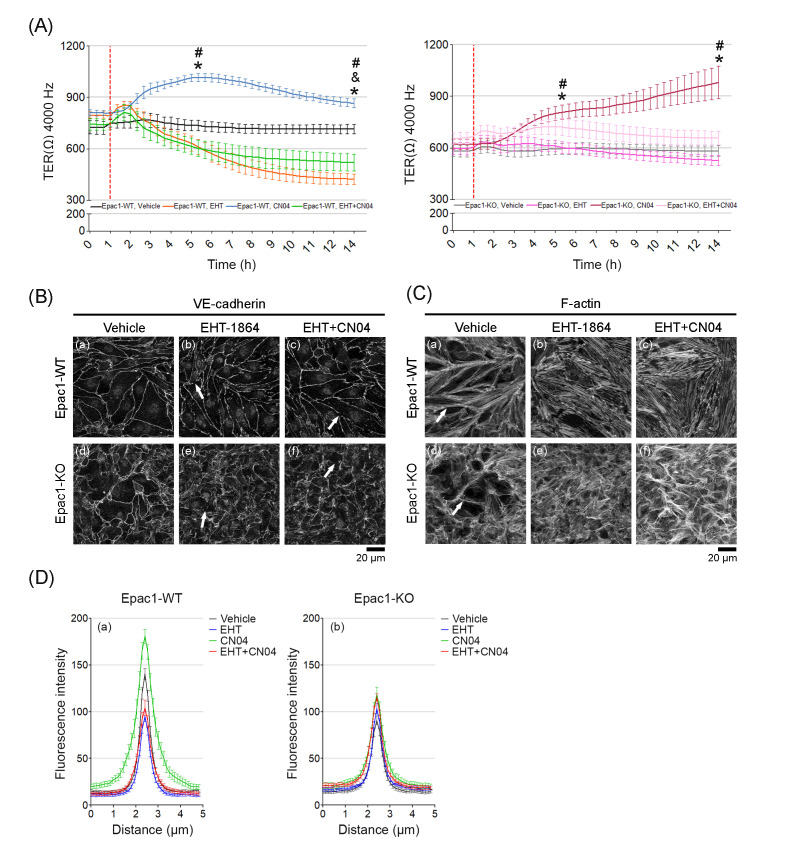
Effects of Rac1 inhibition with EHT-1864 on TER, VE-cadherin, and F-actin. (**A**) Shows the barrier resistance (TER) of confluent WT or Epac1-KO endothelial cell monolayers during 14 h of incubation. After 1 h (segmented red line), the cells received either vehicle (H2O), EHT-1864, or CN04 alone, and EHT-1864 + CN04; N = 5; n = 10 for each condition, “*” shows significance for vehicle vs. the respective CN04 groups; “&” for vehicle vs. EHT-1864; “#” for CN04 vs. EHT-1864. (**B**) Shows VE-cadherin immunostaining of confluent WT and KO cell monolayers; N = 3 for each condition, given either vehicle (a and d), EHT-1864 (b and e), or EHT-1864 + CN04 (c and f). The arrows indicate the sites with disrupted VE-cadherin staining. (**C**) F-actin stained confluent cell sheets treated similar to panel B; N = 3. (**D**) The intensity of VE-cadherin staining from panel B was quantified by densitometric measurements, (a) for WT and (b) for the Epac1-KO cells; N = 3; n = 25 cells per N.

The importance of Rac1 and RhoA for TER was also probed by incubating the confluent WT and Epac1-KO cell monolayers with the Rac inhibitor EHT-1864. The TER remained stable during basal conditions for either cell line (Figure 7, black line for WT and gray line for KO). For the WT cells, CN04 increased (Figure 7A, blue line) and EHT-1864 decreased (Figure 7A, orange line) the resistance, suggesting that both Rac1 and RhoA contributed to the mechanism underlining endothelial barrier integrity. The constitutive Rac1 activity might be more important than an increased RhoA activity, since the decline of TER with CN04+EHT-1864 was nearly as strong as that for EHT-1864 alone (Figure 7A, green line). Surprisingly, Epac1-KO cells showed only a mild reduction in TER, after EHT-1864 administration (Figure 7A, magenta line). To know if the above-mentioned effects could be related to an altered distribution of VE-cadherin or F-actin, the cells were analyzed by immunostaining. After treatment with EHT-1864 or EHT-1864+CN04, either cell type had partially fragmented VE-cadherin immunostaining along cell borders (Figure 7B, b, e, c and f, arrows, respectively). Quantification from the VE-cadherin-mediated contacts revealed that the combined treatment of EHT-1864 with CN04 showed no positive effect in the Epac1-WT cells (Figure 7D, a); whereas the Epac1-KO behaved similar to the CN04 treatments alone (Figure 7D, b). As expected, addition of EHT-1864 in the WT cells led to a complete disruption of the cortical actin ring that is normally associated with increased Rac1 activity [7], which could not be recovered after a CN04 application (Figure 7C, a–c). On the other hand, the actin stress fibers were still present (Figure 7C, c). The CN04-treated Epac1-KO cells showed a cortical-like arrangement of the actin filaments along the junctions together with an elevated stress fiber formation (Figure 7C, e–f), an effect still visible after simultaneous addition of EHT-1864 and CN04. We concluded that once Rac1 function was lost, the endothelial barrier function could be preserved via recruitment of actin stress fibers to the cortical actin ring, through a mechanism that apparently involved RhoA.

## 4. Discussion

Here, we described the establishment of an immortalized cardiac endothelial cell line from WT and Epac1 null mice. The novel cell lines enabled the study of Epac1-deficency on endothelial cells, without the drawbacks of using pharmacological inhibitors or transfections, with anti-sense Epac1 RNA. They also allowed comparison between endothelial cell behavior in vivo and in vitro, since the mice of origin were available. We reported here that Epac1 was required to maintain endothelial barrier function and the integrity of adherens junctions at both the basal state cAMP level and after the strong cAMP stimulation. The deficient barrier function of Epac1-KO cells could not be rescued by cAMP stimulation, indicating that Epac1 is the major mediator of cAMP-induced barrier tightening in the cardiac endothelial cell lines. Rac1 appeared to be a major mediator of the Epac1 action, since Rac1 inhibition only affected the barrier properties in Epac1 expressing cells.

We also found that the activation of RhoA could be required to enhance the endothelial barrier function, since simultaneous strong activation of Rac1 and RhoA resulted in higher resistance. This finding was surprising, since RhoA activation was linked to increased paracellular permeability [7,12,44], but it could explain why cAMP-induced Rac1 activation alone was not sufficient to enhance barrier functions, when Epac1 was absent. We also presented evidence that Epac1 might affect the endothelial cell content of VE-cadherin, Rap1, Rac1, and RhoA, as well as that of cAMP.

### 4.1. Epac1 Is Required for Baseline Integrity and cAMP-Mediated Regulation of Endothelial Adherens Junctions

Our finding that Epac1 was required for proper baseline barrier stability of microvascular myocardial endothelial cells, and for integrity of VE-cadherin-mediated junctions, was in general agreement with studies by Rampersad et al., where in human arterial endothelial cells (HAECs), siRNA-mediated Epac1 knockdown, resulted in compromised endothelial barrier function [41]. Specifically, we found that cells with complete Epac1 loss showed a fragmented VE-cadherin membrane localization, which could explain the reduced baseline TER. The increased junctional leakage of the Epac1 null cells was not related to the loss of VE-cadherin protein. VE-cadherin mRNA was not upregulated in the KO cells, suggesting that post-transcriptional VE-cadherin upregulation might serve as a compensatory mechanism preserving the endothelial AJs and barrier function. However, increased VE-cadherin expression was insufficient to compensate for the loss of Epac1, suggesting that other factors such as molecule delivery at specific subcellular domains in an Epac1-dependent manner might be necessary. We concluded that Epac1 has profound effects on the endothelial barrier function and structure, even at the resting state cAMP level. The findings are in line with the in vivo study reported by Kooperud et al. [3], which showed that in contrast to WT mice, in Epac1-null animals, several organs showed increased microvascular permeability under basal conditions.

A strong cAMP stimulation of the MyEnd cells through a simultaneous application of forskolin and rolipram, increased the barrier resistance further in WT cells, but not in the Epac1-KO cells. When subjected to specific PKA activation by 6-Bnz-cAMP, neither WT nor the KO cells showed increased resistance, while the Epac1 activator 007 caused a discrete but significant increase in resistance, only in the WT cells. We concluded that Epac1 is critically required for cAMP-mediated barrier stabilization in the neonatal myocardial endothelial-derived cells. In contrast, Rampersad et al. found that forskolin or rolipram could counteract the permeability increase caused by Epac1 knockdown in adult human aorta endothelial cells. A possible explanation for the discrepancy could be that aorta endothelial cell junctions are differently regulated. They are not involved in intercellular transport, like micro-vascular endothelial cells are. Additionally, Rampersad et al. used siRNA to reduce the expression of Epac1, which might not ensure a complete absence of Epac1 under all conditions.

Our findings confirmed the importance of Epac1 for baseline and cAMP-mediated barrier regulation. However, Epac1-independent mechanisms can also contribute. Several studies point to PKA as an important mediator of cAMP-induced endothelial barrier stabilization [36,45,46]. We found that specific activation of PKA was insufficient to enhance TER (Figure 1B and Supplementary Figure S1).

We found that the endothelium lacking Epac1 showed an increased expression of Epac2 (Supplementary Figure S6). However, the expression of Epac2 is unlikely to affect trans-endothelial resistance, since Epac2 null mice have WT-like micro-vascular permeability [3]. It should also be noted that Epac1 and Epac2 have different subcellular localization [19,47], therefore, it is unlikely for Epac2 to have a direct effect near the endothelial surface membrane. Moreover, it is known that activation of Epac2 in vivo can contribute to arrhythmia and hampered cardiac function [48,49]. Apparently, the main role of Epac2 is to control oxidative processes, as seen during liver regeneration [50]. We speculate that the slight Epac2 expression could be mediated through Epac2 transcription factors activated by signals originating as a consequence of weakened cell–cell contacts.

### 4.2. Epac1 Is Required for cAMP-Mediated Rap1, but Not for Rac1 and RhoA Activity

It is commonly thought that increased Rap1/Rac1 activity is associated with enhancement of VE-cadherin-based junction resistance and of cortical actin stability, while activation of RhoA usually correlates with increased paracellular permeability and enhanced actomyosin contractility [7,12,44]. We found that Rac1, RhoA, and Rap1 protein levels were higher in our Epac1-null cells at basal state (Figure 3 and Figure 4). Still, the total Rac1 and RhoA baseline activity (Figure 4C) was similar in either cell line. The Rap1 activity was far lower in KO than in WT cells, both at basal state and after cAMP-stimulation (Figure 3) but could still be triggered. On the other hand, cAMP stimulation resulted in similar Rac1 activation in both WT and Epac1-KO cell lines (Figure 4D). These results suggest that increased cAMP might lead to increased Rap1/Rac1 activation in an Epac1-independent manner. One candidate is the phosphoinositide-specific phospholipase C (PLCε), which was shown to stimulate Rap1/Rac1 activity after cAMP increase via KRIT1 [51]. Altogether, Epac1 appeared to be required for proper baseline and cAMP-mediated activation of Rap1 but might be less important for Rac1 activation. 

Epac1 might also enhance adhesion by promoting the proper translocation of Rho GTPase-specific GEFs to the relevant regions of the membrane. This possibility is supported by evidence that the Epac1 effector Rap1, by binding Rac1 GEFs, Vav2, and Tiam1, could drive cell spreading in HeLa cells [52]. Furthermore, Vav2 and Tiam1 are known to exert protective barrier effects in human endothelial pulmonary cells through a mechanism involving prostaglandin E2 and Epac1/Rap1/Rac1 signaling [45]. There is also evidence that Epac1/Rap1 via the effector Rasip1 could interact with the RhoA-GAP ArhGAP29 and thereby regulate RhoA activity to strengthen the endothelial barrier [53]. Thus, several potential interactions between Epac1 effectors and respective GEFs for Rac1 and RhoA could exist and might be important for barrier regulation, some of which, are discussed elsewhere [54].

### 4.3. Epac1 Deletion Might Regulate cAMP Levels in a PDE4-Dependent Manner

An increase of endothelial cell cAMP levels is usually associated with the strengthening of the endothelial cell barrier [3,25,34,46,55,56]. We found that endothelial cells lacking Epac1 showed higher levels of cAMP and PDE4-D, but slightly lower net baseline PDE activity (Figure 5). We speculate that the discrepancy between increased PDE4-D expression and increased cAMP level might be related to the finding described by Rampersad et al. [41] in aortic endothelial cells, whose PDE4-D was tethered by Epac1 in a VE-cadherin signaling complex, with PKA, within a sub-membranous compartment. This arrangement promoted activatory PKA-mediated phosphorylation of PDE4-D, when cAMP rose in the compartment, thus, producing a local negative feedback loop for the compartment cAMP concentration. If a similar situation existed in our endothelial cells, the deletion of Epac1 would translocate PDE4-D from the sub-membranous compartment to the general cytoplasm. This would lead to an increased local cAMP level and PKA activity in the sub-membrane compartment. It might also increase bulk cAMP in the Epac1 null cells, since the translocated PDE4-D would be less exposed to activating phosphorylation through PKA, after translocation, than when juxtaposed to PKA in the submembrane compartment. This might be one explanation why our Epac1 null cells showed higher cAMP levels than the WT cells.

### 4.4. Roles of Rac1 and RhoA for Epac1-Mediated Barrier Regulation

The primary mechanism underlying defective barrier function under basal conditions in the current endothelium model appeared to be impaired Rap1 activation. This could be concluded from the fact that, despite the higher expression of Rap1 in Epac1-KO cells, the net activity appeared to be decreased (Figure 3C). In addition, the Rac1 inhibitor EHT-1864, which reduced the barrier function in WT cells, did not affect the barrier properties of the Epac1-deficient cells. In contrast, Rac1 inhibition in WT cells resulted in partially fragmented VE-cadherin staining and complete disruption of cortical actin, paralleled by lower TER. These results suggest that, at least to some extent, other GTPases or GEFs might be able to compensate the lack of Epac1. This is specially supported by the fact that cells lacking Epac1 showed a more defined cortical actin, across all conditions tested.

The novel synthetic direct GTPase activator CN04, which directly activates both Rac1 and RhoA, was able to bypass Epac1 loss, and enhanced the activity of Rac1 and RhoA in WT and Epac1-KO cells. In the Epac1 null cells, treatment with CN04 induced a delayed but constant TER increase, which reached a very similar level to the one hit by the CN04-treated WT cells. Moreover, in either cell line, application of CN04 widened the VE-cadherin-mediated junctions and enhanced the localization of the F-actin bundles at the cortical actin ring, as observed by immunostaining analyses. These findings indicate that RhoA activation might enhance the barrier properties of MyEnds or that, at the very least, concerted action between Rac1 and RhoA is required. These findings are in contrast to the well-documented endothelial barrier dysfunction caused by RhoA activation [2,42,44,57]. Some reports by other groups do support that RhoA activity might promote barrier stability. In this context, Zhang et al., taking advantage of the recently developed FRET biosensors for GTPases, described that S1P-treated HUVECs showed early local activation of RhoA at the cell periphery, leading to a cortical actin ring reinforcement and myosin light chain phosphorylation, in a Rac1-independent manner [43]. Another example underlining the protective effects of RhoA is the elegant study by Heemskerk et al., demonstrating that endothelial vascular leak during leukocyte migration across endothelial vessels is prevented by local activation of RhoA-mediated actin contractility [58].

Altogether, these results highlight that RhoA activity might be required for cAMP-mediated barrier stabilization and could partially compensate for the loss of Epac1 in myocardial microvascular endothelial cells.

## 5. Conclusions

We present two novel cardiac endothelial cell lines, one derived from WT and one from Epac1 null mice. These demonstrate that Epac1 deficiency leads to morphologically weaker cell junctions with a lower content of VE-cadherin. As expected from studies using Epac1 knockdown techniques or pharmacological manipulation [59,60], the Epac1 null endothelial cells also showed less Rap1 activity and increased barrier leakage. They allowed us to also show that the Epac1-deficent barrier could be restored by simultaneous activation of Rac1 and RhoA, but not by Rac1 activation alone. The activation of RhoA, primarily associated with barrier dysfunction through enhanced actomyosin contractility [7,12,44], might therefore also promote endothelial barrier tightening. 

The Epac1-deficient cell monolayers showed several apparently compensatory features, like augmented cAMP content, and increased expression of VE-cadherin, Rap1, Rac1, and RhoA proteins. However, the finer regulatory details pertaining to the expression of such proteins remain to be elucidated in future studies.

Overall, our results emphasize the importance of Epac1 for maintenance of endothelial barrier function and demonstrate that part of the Epac1 effect might be mediated by regulation of not only Rap1, but also involve Rac1, and RhoA.

## Figures and Tables

**Table 1:** Primer sequences.

mEpac1-378bp-FW	CAGGTCAGCGTACGGATGAAGAAC
mEpac1-378bp-REV	GCTTCCACATCCTTGATGATGCG
mVE-cadherin-139bp-FW	CTGTCTTCCAGCGACACTTCTAC
mVE-cadherin-139bp-REV	GCCTCTGTCACTGGTCTTGC
mB2M-292+788bp-FW	CAAGTATACTCACGCCACCCAC
mB2M-292+788bp-REV	CATCATGATGCTTGATCACATGTCTC

## Supplementary Materials

The following are available online at https://www.mdpi.com/2073-4409/9/10/2170/s1, Figure S1: Barrier function over time measured by ECIS, Figure S2: Representative Western blot of pVASP and total VASP from confluent WT and Epac1-KO cell monolayers, Figure S3: Original Western blot images, Figure S4: mRNA expression analysis in WT and Epac1-KO cells, Figure S5: Rap1 expression in WT and Epac1-KO cells, Figure S6: Epac2 expression in WT and Epac1-KO cells, Figure S7: PDE4-D expression in WT and Epac-KO cells, Table S1: Summary of TER modeling.

## Abbreviations

**Abbreviations**	**Nomenclature**
**AJ**	Adherens junction
**AC**	Adenylyl Cyclase
**CNF-1**	Cytotoxic necrotizing factor 1
**cAMP**	Cyclic adenosine monophosphate
**Epac1**	Exchange protein activated by cAMP 1
**GEFs**	Guanosine nucleotide exchange factors
**GTPases**	Guanosine triphosphatases
**G-LISA**	G-protein ELISA
**HUVEC**	Human Umbilical Vein Endothelial Cells
**HDMEC**	Human Dermal Microvascular Endothelial Cells
**MyEnds**	Myocardial endothelial cells
**PKA**	Protein Kinase A
**PDE**	Phosphodiesterase
**TER**	Transendothelial electrical resistance
**VASP**	Vasodilator-stimulated phosphoprotein
**007**	8-pCPT-2′-O-Me-cAMP
