# Supplementary material for: Epac1 Is Crucial for Maintenance of Endothelial Barrier Function through A Mechanism Partly Independent of Rac1
Source: Cells. 2020 Sep 25;9(10):2170. doi: 10.3390/cells9102170 (PMC7601253; doi:10.3390/cells9102170)
Supplement: Supplementary file 1 [file cells-09-02170-s001.pdf]

Rb		Alpha		Cm	
Epac1-WT	Epac1-KO	Epac1-WT	Epac1-KO	Epac1-WT	Epac1-KO
0.7225 ± 0.02558	0.3983 ± 0.02132	4.110 ± 0.05772	3.302 ± 0.06047	0.6475 ± 0.04566	0.3925 ± 0.04459

Table S1 Summary of TER modeling. Rb, represents the real barrier function. α, describes the space between the cells and the matrix. Cm, depicts the membrane capacitance. Data are presented as Mean ± SEM. n=4

(A)

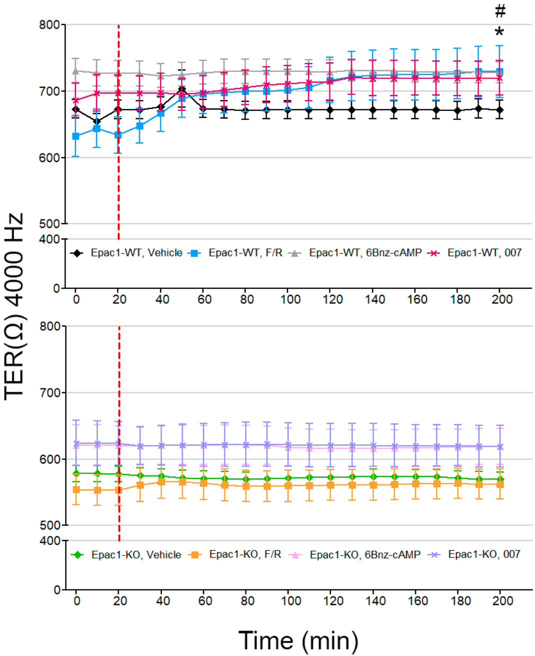

Figure S1 Barrier function over time measured by ECIS. (A) TER in control and treated WT and Epac1-KO cell monolayers.Segmented red line denotes time of analogs application. Data are presented as mean ± SEM.N = 4-5;n ≥ 8. " * " represents significant difference between WT-vehicle and WT-F/R. " # " represents significant difference between WT-vehicle and WT-007.

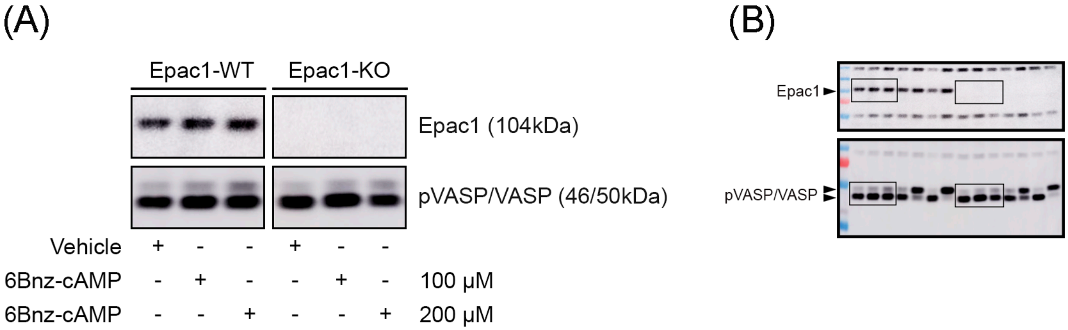

Figure S2 Representative Western blot of pVASP and total VASP from confluent WT and Epac1-KO cell monolayers (A) treated with either vehicle, 100 μM or 200 μM of 6Bnz-cAMP as indicated. Expression of Epac1 is also shown as internal control, N = 6 (B) Original uncropped gel images depicted in (A), arrowheads indicate bands of interest, black line frames show the bands used for all representative images

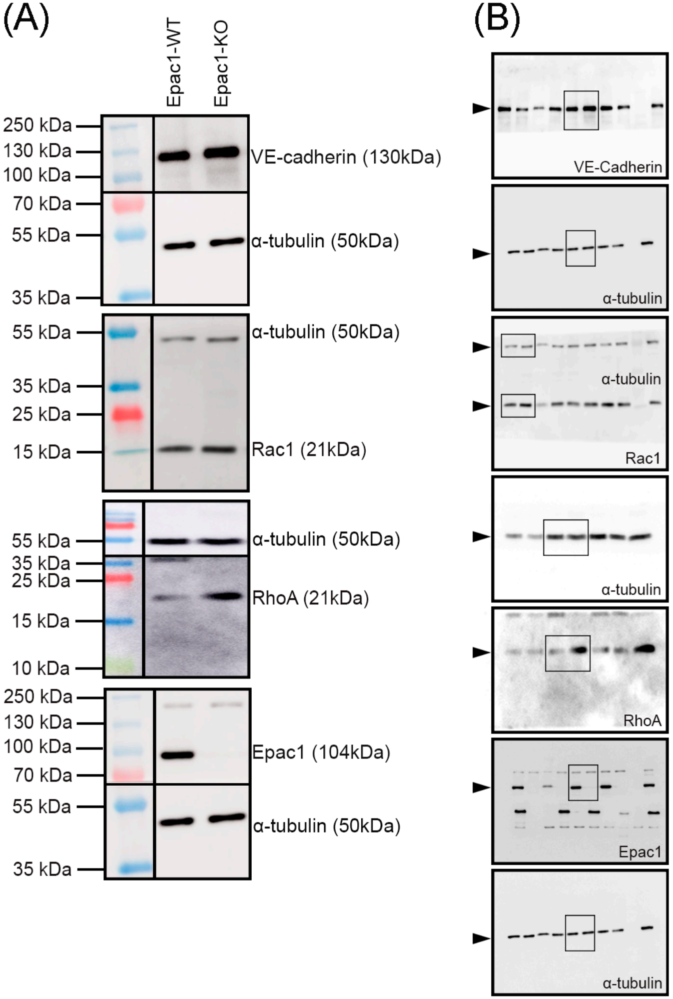

Figure S3 Original Western blot images. (A) Cropped blots presented in Figures 2 and 4 merged with corresponding colorimetric molecular weight marker.
(B) Full-length Western blots from the cropped membranes presented in (A). Arrowheads indicate bands of interest; black line frames indicate the cut sections.

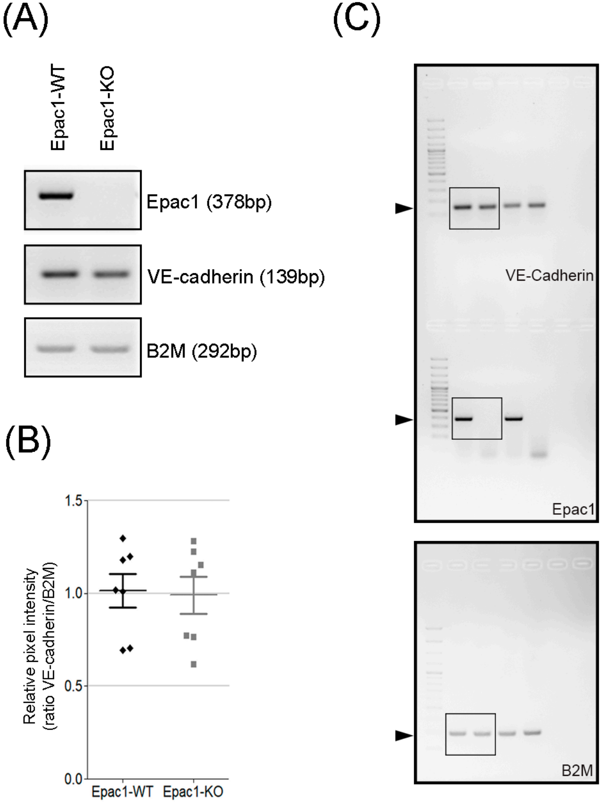

Figure S4 mRNA expression analysis in WT and Epac1-KO cells. (A) Analysis of VE-cadherin mRNA expression by RT-PCR. Lack or presence of Epac1 was confirmed by gene-specific primers. Amplification of B2M was used to verify equal loading; n≥ 8. (B) Densitometric measurements of the band intensity from obtained amplicons determined the relative mRNA expression of VE-cadherin normalized to B2M. (C) Original uncropped gel images depicted in (A), arrowheads indicate bands of interest, black line frames show the bands used for all representative images; n≥ 8.

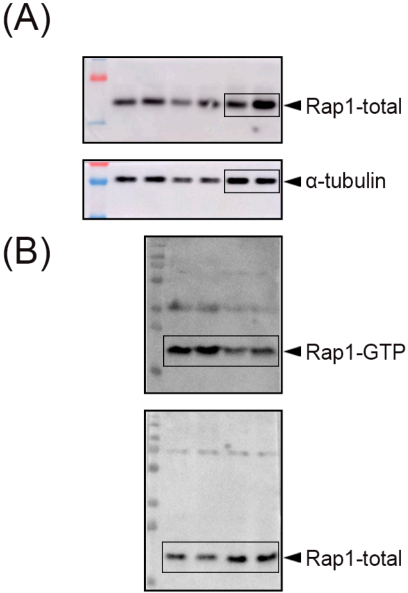

Figure S5 Rap1 protein expression in WT and Epac1-KO cells. (A) Original Western blot images from basal Rap1 and (B) Rap1-GTP pulldown assays presented in Figure 3. Arrowheads indicate bands of interest, black line frames indicate cropped sections.

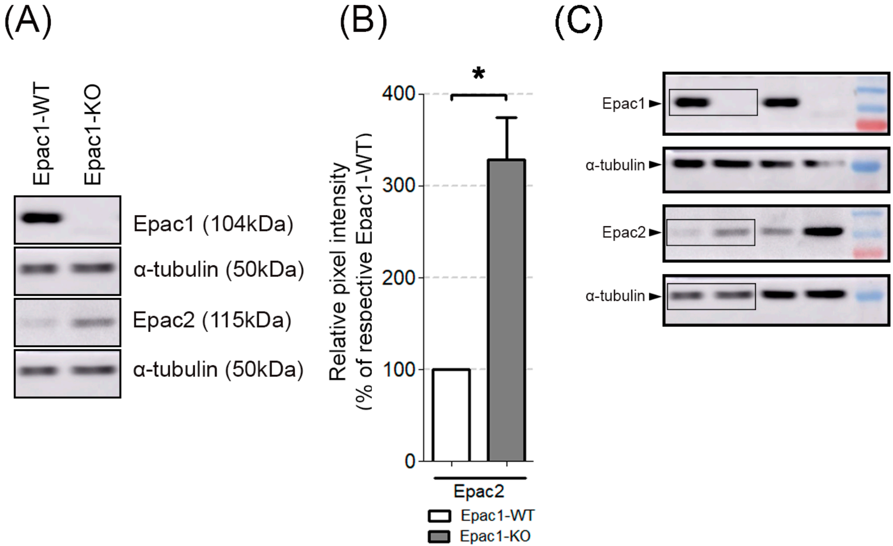

Figure S6 Epac2 expression in WT and Epac1-KO cells. (A) Representative Western blot from Epac1 and Epac2, α-tubulin was used as loading control. (B) Densitometric measurements from blots presented in (A); N=6 (C) Original Western blot images from (A). Arrowheads indicate bands of interest, black line frames indicate cropped sections.

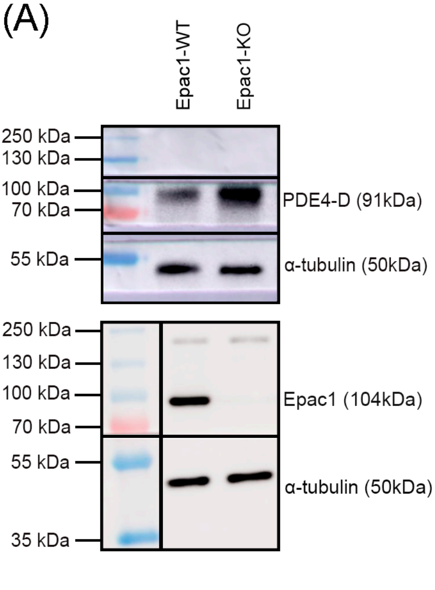

Figure S7 PDE4-D expression in WT and Epac1-KO cells. (A) Cropped Western blot images displayed at Figure 5 merged with corresponding colorimetric molecular weight marker. (B) Full-length Western blots of the cropped membranes presented in (A). Arrowheads indicate bands of interest; black line frames indicate the cut sections.
